# Comparing antimicrobial resistant genes and phenotypes across multiple sequencing platforms and assays for *Enterobacterales* clinical isolates

**DOI:** 10.1186/s12866-023-02975-x

**Published:** 2023-08-18

**Authors:** Rebecca Rose, David J. Nolan, Deborah Ashcraft, Amy K. Feehan, Leonor Velez-Climent, Christopher Huston, Benjamin Lain, Simon Rosenthal, Lucio Miele, Gary B. Fogel, George Pankey, Julia Garcia-Diaz, Susanna L. Lamers

**Affiliations:** 1 BioInfoExperts LLC, 718 Bayou Lane, Thibodaux, LA 70301 USA; 2 FoxSeq, LLC, Thibodaux, LA USA; 3 Infectious Disease Translational Research, Ochsner Clinic Foundation, New Orleans, LA USA; 4 Infectious Disease Clinical Research, Ochsner Clinic Foundation, New Orleans, LA USA; 5 Translational Science and Genetics at Louisiana State University Health Science Center, New Orleans, LA USA; 6 Natural Selection, Inc., CA San Diego, USA

**Keywords:** Carbapenem, *blaKPC*, MicroScan, ETEST, Assembly methods, Anti-microbial drugs, *Klebsiella*, *Enterobacter*

## Abstract

**Introduction:**

Whole genome sequencing (WGS) of bacterial isolates can be used to identify antimicrobial resistance (AMR) genes. Previous studies have shown that genotype-based AMR has variable accuracy for predicting carbapenem resistance in carbapenem-resistant *Enterobacterales* (CRE); however, the majority of these studies used short-read platforms (e.g. Illumina) to generate sequence data. In this study, our objective was to determine whether Oxford Nanopore Technologies (ONT) long-read WGS would improve detection of carbapenem AMR genes with respect to short-read only WGS for nine clinical CRE samples. We measured the minimum inhibitory breakpoint (MIC) using two phenotype assays (MicroScan and ETEST) for six antibiotics, including two carbapenems (meropenem and ertapenem) and four non-carbapenems (gentamicin, ciprofloxacin, cefepime, and trimethoprim/sulfamethoxazole). We generated short-read data using the Illumina NextSeq and long-read data using the ONT MinION. Four assembly methods were compared: ONT-only assembly; ONT-only assembly plus short-read polish; ONT + short-read hybrid assembly plus short-read polish; short-read only assembly.

**Results:**

Consistent with previous studies, our results suggest that the hybrid assembly produced the highest quality results as measured by gene completeness and contig circularization. However, ONT-only methods had minimal impact on the detection of AMR genes and plasmids compared to short-read methods, although, notably, differences in gene copy number differed between methods. All four assembly methods showed identical presence/absence of the *blaKPC-2* carbapenemase gene for all samples. The two phenotype assays showed 100% concordant results for the non-carbapenems, but only 65% concordance for the two carbapenems. The presence/absence of AMR genes was 100% concordant with AMR phenotypes for all four non-carbapenem drugs, although only 22%—50% sensitivity for the carbapenems.

**Conclusions:**

Overall, these findings suggest that the lack of complete correspondence between CRE AMR genotype and phenotype for carbapenems, while concerning, is independent of sequencing platform/assembly method.

**Supplementary Information:**

The online version contains supplementary material available at 10.1186/s12866-023-02975-x.

## Background

Whole genome sequencing (WGS) of bacteria is used increasingly for retrospective molecular epidemiology of bacteria, including species classification, identifying genetically related strains that are part of outbreaks [1–5], and predicting antimicrobial resistance (AMR). WGS has advantages over standard antimicrobial susceptibility testing procedures, such as the ability to detect novel AMR genes [1], and its applicability for any pathogenic bacterial species [6]. WGS can also, in theory, more precisely identify the mechanism of underlying resistance and accurately assess multidrug-resistance (MDR) [7].

WGS has excellent potential to become a powerful diagnostic tool in hospital settings [8–10], particularly for epidemiological questions [9, 11]. However, while the clinical adoption of WGS-based methods is theoretically exciting, this application of WGS presents more hurdles, including the time required to generate, process, and interpret AMR genotypes compared to standard microbial phenotypic assays; the technological infrastructure required to integrate WGS results with patient data; and the non-straightforward relationship between AMR genotypes and phenotypes. While first two hurdles are being actively addressed through improved technology, the latter hurdle is less straightforward to overcome. AMR is a complex phenomenon, and there are multiple reasons why the presence/absence of an AMR gene may correspond imperfectly with microbial phenotypic assays, including experimental (e.g., low quality samples [12]; differences in sequencing platforms/pipelines [13]; incomplete comparative databases [12]); clinical (e.g., interpretations of phenotypic tests may vary among institutions and/or over time due to changes in breakpoints or assays), and/or biological (e.g., variation among strains [14], polygeny and pleiotropy [14]; variability in gene expression with respect to environmental conditions [15]). Furthermore, both genotype-based and phenotype-based AMR assays do not account for other factors that can influence actual patient outcomes, including host response, interactions with other microorganisms, and bioavailability in tissue [16]. All of these factors need to be considered for WGS-based methods to become part of the standard of care.

One important aspect of WGS is the choice of sequencing platform, which generally can be categorized as short-read or long-read technology [17]. Short-read sequencing, which produces sequences or “reads” < 500 nucleotides (*nt*) in length, is dominated by Illumina platforms. While short reads have high base-calling accuracy, de novo assembly of genomic regions with high numbers of repeats and/or small extra-chromosomal sequences, such as mobile genetic elements (MGE), remains challenging [18]. Since AMR genes are associated frequently with MGE [19], inaccurate reconstruction could impact downstream analyses. Furthermore, the copy number of AMR genes may impact bacterial phenotype [20]. Therefore, identifying the presence and the quantity of AMR genes accurately is essential towards AMR genomic testing in clinical settings. Long-read sequencing (> 1,000 nt reads) technologies are dominated by two platforms: Oxford Nanopore Technologies (ONT) [21] and Pacific Biosciences single molecule, real-time sequencing [22]. Long reads are advantageous because they are more likely to reconstruct repeat regions and are more likely to generate circular or closed genomes and plasmids [23]). However, although the technology is improving, long-read technologies are associated with higher rates of base-calling errors [24], are more expensive to generate, and require greater computational resources. A third option is a hybrid approach, which combines short and long reads for de novo assembly and AMR prediction, thereby combining the advantages of both approaches [18, 25–32]. However, this is the costliest option, and therefore may not be practical currently for routine clinical settings. Furthermore, this approach requires additional time for sequencing and presents bioinformatics hurdles, which reduces its value in microbiology diagnostic workflows.

In a previous study, we used short-read WGS on 51 carbapenem-resistant *Enterobacterales* (CRE) isolates (Rose et al*.*, submitted) to compare genotype-based classification and AMR prediction with matrix-assisted laser desorption/ionization time-of-flight (MALDI-TOF) mass spectrometry for classification and automated MicroScan AMR for AMR phenotyping. We found that the presence/absence of β-lactamase genes (i.e. *blaKPC*) correlated with β-lactam susceptibility as assessed by MicroScan with only 78% sensitivity and 89% specificity for meropenem, and only 56% sensitivity for ertapenem (all isolates were non-susceptible), and the inclusion of other AMRs with potential to impact β-lactam susceptibility (i.e. porin genes, *blaCTX-M-15*), failed to account for the discrepancy. To investigate whether these inconsistencies were due to incomplete genomic coverage resulting from short-read sequencing, we selected nine previously analyzed CRE isolates from three species (*Klebsiella pneumoniae, Klebsiella aerogenes,* and* Enterobacter cloacae complex*) for long-read ONT WGS, which had been shown to out-perform Pacific Bioscience in this use case as well as provide significant cost-savings [30]. Our goal was to determine whether long-read sequencing would resolve the discordance previously found between the presence/absence of β-lactamase genes (i.e. *blaKPC*) and β-lactam susceptibility, and if so, whether long-read sequencing alone was sufficient, or whether the combination of both long and short reads was required. We also performed a second manual phenotype test (ETEST) to increase confidence in using the automated phenotype AMR result as the gold standard.

## Results

### Coverage

Overall, the median number of ONT raw reads across the nine samples was 314,906; and the median number of nucleotides sequenced was 1.2 M, resulting in a median coverage of 242x (calculated as the number of total base pairs/the estimated length of the genome). Overall, ONT provided 1.1x – 3.9x coverage as compared to Illumina (Supplemental Table 1).

**Fig. 1:**
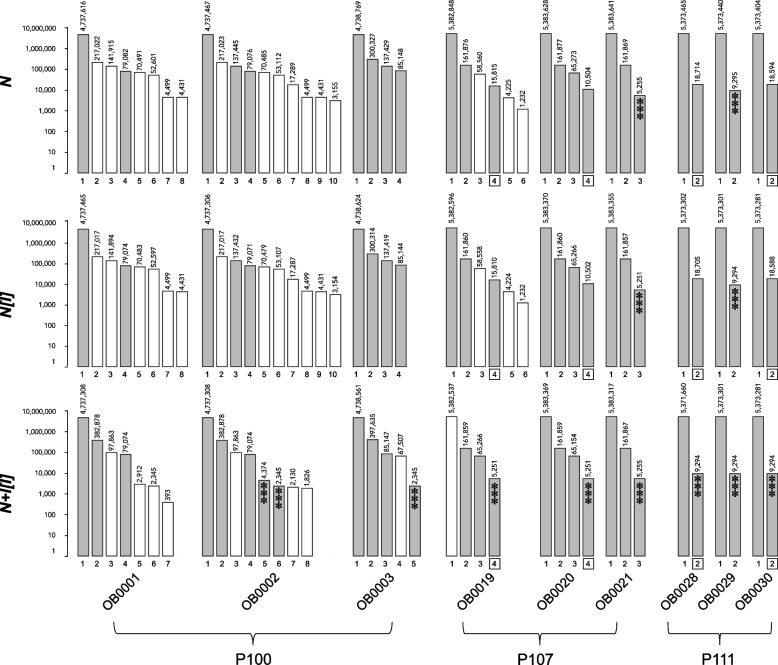
Bar chart of contig IDs (x-axis) and lengths (y-axis, log scale) for long-read sequence data using three different assembly methods (top, middle, bottom, defined in text). Grey bars indicate a circular/closed contig, open bars indicate a non-circular contig. Lengths in nucleotides are shown above each contig. Three asterisks indicate that a contig of similar length (< 50nt difference) was also found with the short-read only method. Boxed contig numbers indicate a suspected duplication

Four different assembly pipelines were used to align reads to produce longer contiguous sequences (contigs; see Methods for additional details): short-read only (“*I*”); ONT-only (“*N*”); ONT-only followed by a short-read polish (“*N[I]*”); hybrid ONT and short-read followed by a short-read polish (“*N* + *I[I]*”). All three long-read assembly methods resulted in 2–10 contigs per sample (Fig. 1), compared to 42 – 132 contigs per sample for the short-read-only assembly (Supplemental Table 2). On average, the *N* + *I[I]* method resulted in fewer contigs (*n* = 4.1) than *N* or *N[I]*; (*n* = 4.6 for both; Fig. 1). The *N* + *I[I]* method also resulted in more circularized/closed contigs on average (*n* = 3.1) than *N* or *N[I];* (*n* = 2.8 for both).

All three methods using ONT reads resulted in a contig for each sample between 4.3 M – 5.4 M *nt*, consistent with the chromosomal contig (Supplemental Table 2, Fig. 1). With only one exception (OB0019, *N* + *I[I]*), the chromosomal contigs produced by all three methods were circular, and the length difference among the three methods was < 2000 *nt*.

In five isolates (OB0020, OB0021, OB0028, OB0029, OB0030), all three long-read methods resulted in the same number of extra-chromosomal contigs (1 – 3), all of which were closed. In the other four isolates, the difference in the number of extra-chromosomal contigs among methods ranged from 1 – 2.

Interestingly, in three isolates (OB0020, OB0028, OB0030), the shortest contig was ~ 2x the length in the *N* and *N[I]* methods relative to the *N* + *I[I]* method (and *I* method), within a range of 0 – 126 *nt*. In isolate OB0019, the extra-chromosomal contigs found by the *N* + *I[I]* methods were not completely congruent to those found by the other two methods; however, a short closed contig found by *N* + *I[I]* was 3x as long as a closed contig found by the other two methods, within a range of 0 – 165 *nt*. For the remaining three isolates, only one closed extra-chromosomal contig was of similar length among all three methods, ranging in length from 79,071 – 79,076 *nt* (OB0001, OB0002) or 85,144 – 85,148 *nt* (OB0003), and none of the contigs appeared to be obvious duplications or triplications.

### Gene completion

A total of 440 *Enterobacterales* genes were considered by BUSCO. On average, the *N* method found slightly fewer (96.9%) complete, single-copy genes compared to the other three methods (all averaged 98.5%; Supplemental Table 3). For each sample, all methods identified the same number of complete, duplicated gene copies for each sample (range: 1 – 3). For the *N* method, the average number of fragmented (*n* = 5) and missing (*n* = 6.5) genes was higher than the average for the other three methods, for which the average number of fragmented genes ranged from 0.4 – 0.8, and the average number of missing genes ranged from 4.0 – 4.2.

### AMR gene detection

**Fig. 2:**
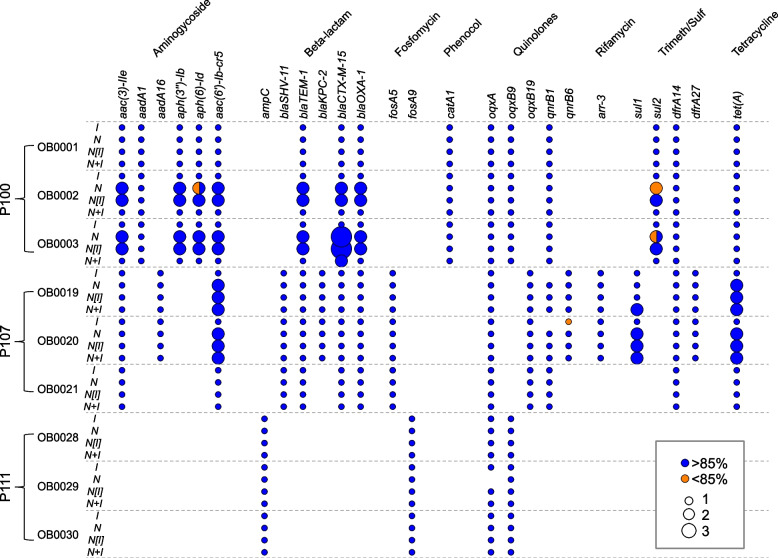
AMR genes were detected in 10 isolates by 4 different assembly methods (defined in text). Circles indicate the presence of the gene, color indicates percent identity to the reference gene, and size is proportional to the number of copies of a gene detected

We then compared all of the acquired AMR genes identified by AMRF for all four assembly methods (Fig. 2). A total of 27 AMR genes were identified over all samples. Of the 109 instances in which a gene was identified for a particular sample, only in 5 cases (4.6%) did one of the methods fail to detect the gene at ≥ 85%. In two of those cases, the *I* method failed to detect the gene at all  (*qnrB1* in OB0019 and OB0020), and in one case, the *N* method failed to detect the gene at all (*oqxA* in OB0029). In one other case (*qnrB6* for OB0020), the *I* method detected the gene, but only with 70% coverage, and in another case (*sul2* for OB0002) the *N* method detected two copies of the gene, both with 70% coverage.

A larger discrepancy among methods was found for the number of copies of the gene. In 22 cases (20%), while all four methods detected the gene, the number of copies varied from 1 – 3. Among the P100 isolates, in samples OB0002 and OB0003 eight genes (*aac3-IIe, aph3″-Ib, aac6’-Ib-cr5, blaOXA-1, blaCTX-M-15, blaTEM-1, aph6-Id*, and *sul2*) were identified as a single copy by two methods (*I, N* + *I[I]*) and in duplicate or triplicate by the other two methods (*N, N[I]*). Among the P107 isolates, in samples OB0019 and OB0020, two genes (*aac6-Ib-cr5, tet(A))* were identified as a single copy by the *I* method, and in duplicate by the other three methods. A third gene (*sul2*) was identified as a single copy by the *I* method and in duplicate by the other three methods for sample OB0019, and in duplicate by the *N* + *I[I]* method and as a single copy by the other three methods for sample OB0020. None of the AMR genes were found on contigs that varied in length by a factor of two or three among methods (Supplemental Table 4). In subsequent analyses we used the set of AMR genes detected by the *N* + *I[I]* method.

### Plasmids

The number of plasmids identified in each isolate based on > 90% replicon sequence identity ranged from 1 – 6 for the long-read assemblies, and 1 – 5 for the short-read only assemblies (Supplemental Table 5). All long-read assemblies identified the same plasmids. Twenty-five plasmids were identified in both the short and long read assemblies over all nine samples. The Col(pHAD28) plasmid was identified by only the long-read assemblies in five samples, although with identity of 92%-93%. In addition, the Col440II plasmid was identified in sample OB0019 at 100% identity in the short-read-only assembly, but in none of the long-read assemblies.

### Localizing AMR genes

**Fig. 3:**
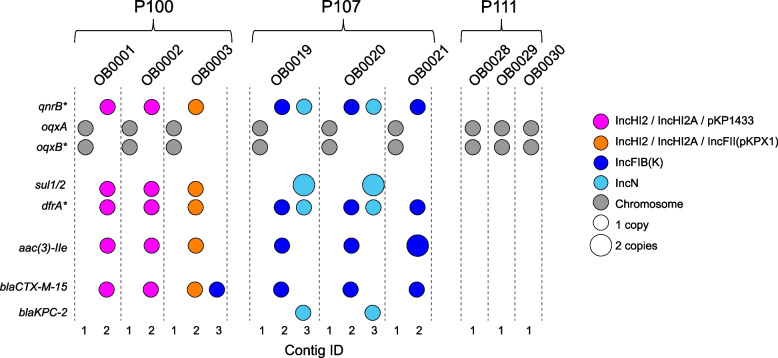
Location of AMR genes detected by the *N* + *I[I]* method. Colors correspond to plasmids found on the same contig as the gene, and size is proportional to the number of copies of a gene detected. Contig ID corresponds with contigs in Fig. 1

We then considered the contigs where AMR genes were found in conjunction with their association with plasmids (Fig. 3). AMR phenotypes were classified for six antimicrobials, including two carbapenems (ertapenem [ETP], meropenem [MEM]) and four non-carbapenem drugs (cefepime [FEP], trimethoprim/ sulfamethoxazole [T/S], gentamicin [GEN], and ciprofloxacin [CPFV]); therefore, we specifically considered the set of genes relevant to these drugs as follows: CPFV: *qnrB, oqxA,oqxB*; T/S: *sul1,sul2,* and variants (*) of drfA*; GEN: aac*3-IIe; FEP: *blaCTX-M-1*; ETP/MEM: *blaKPC-2*. All isolates had the chromosomal *oqxA* and *oqxB* genes. None of the isolates from P111 had extra-chromosomal AMR genes. For P100, all three isolates (OB0001, OB0002, and OB0003) shared five AMR genes located on contig #2. In all three isolates, this contig was associated with plasmids IncHI2 and IncHI2A, plus pKP1433 (in OB0001 and OB0002) or IncFII (in OB0003). OB0003 also had a second copy of *bla*CTX-M-15 on contig #3, associated with plasmid IncFIB(K). For P107, all three isolates shared four AMR genes on contig #2 associated with the IncFIB(K) plasmid. Two isolates (OB0019, OB0020) also shared an additional four AMR genes (including the *blaKPC-2* gene) located on contig #3, associated with the IncN plasmid and the transposon Tn4401a sequence.

### Concordance between AMR genotypes/phenotypes

**Fig. 4:**
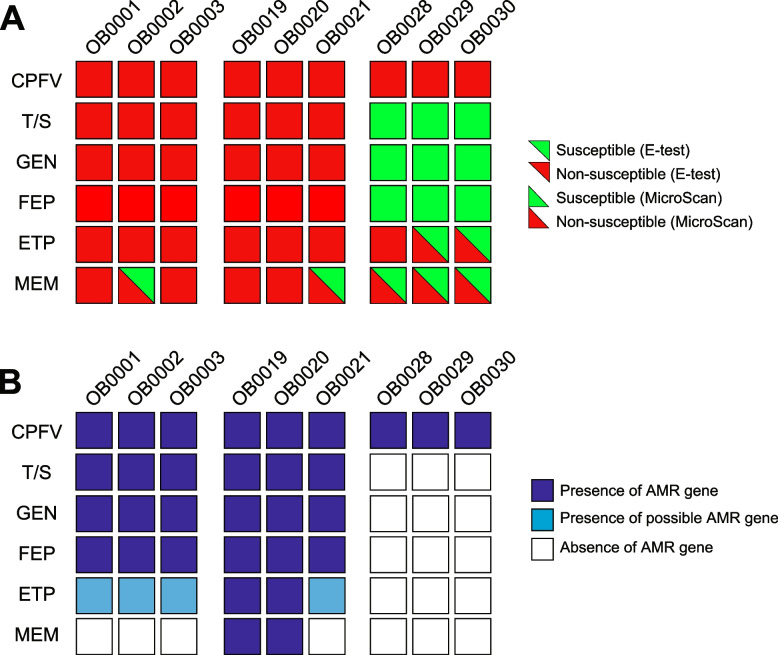
Phenotype and genotype results. **A** Phenotype AMR for MicroScan and ETEST (bottom triangle/upper triangle, respectively, according to the color legend). **B** Presence/absence of AMR genes corresponding to the drugs tested in (**A**) according to the legend. CPFV = ciprofloxacin; T/S = trimethoprim/sulfamethoxazole; GEN = gentamicin; FEP = cefepime; ETP = ertapenem; MEM = meropenem

Two phenotype assays were used (MicroScan and ESTEST) to assess resistance to the six drugs (Fig. 4A, Table 1). For the four non-carbapenem drugs (CPFV, T/S, GEN, FEP), the initial MicroScan and subsequent ETEST results were 100% concordant. On the other hand, the two phenotype assays showed discordant results in 7/20 (35%) tests for the two carbapenem drugs (ETP/MEM). In all cases, the ETEST showed the sample as susceptible, while the MicroScan test showed the sample as non-susceptible.

The presence/absence of the AMR genes for the four non-carbapenem drugs also corresponded exactly to the resistance profile in all ten samples (i.e., presence/absence of *qnrB, oqxA/oqxB* [CPFV]; sul1/sul2/drfA* [T/S]; aac*3-IIe [GEN]; *blaCTX-M-1* [FEP]; Fig. 4B). For the ETEST results, presence of the *blaKPC-2* gene showed 100% specificity for both drugs, although only 29% and 50% sensitivity to ETP and MEM, respectively (Fig. 4B; Table 1). For the MicroScan results, presence of *blaKPC-2* showed only 22% sensitivity to ETP and MEM (all samples were phenotypically non-susceptible to both drugs). When considering the *blaCTX-M-15* gene as a possible contributor to ertapenem resistance, the combined presence of *blaKPC-2* and *blaCTX-M-15* increased sensitivity to the ETEST and MicroScan to 86% and 67%, respectively.

**Table 1:** AMR genotype–phenotype concordance for six drugs

Drug	Test	Genes	GR/ PR	GS/ PR	GR/ PS	GS/ PS	SN	SP
CPFV	Both	oqxA; oqxB*,qnrB	9	0	0	0	1.00	NA
T/S	Both	sul1 or sul2. dfrA*	6	0	0	3	1.00	1.00
GEN	Both	*aac(3)-IIe*	6	0	0	3	1.00	1.00
FEP	Both	*blaCTX-M*	6	0	0	3	1.00	1.00
ETP	ETEST	blaKPC*	2	5	0	2	0.29	1.00
MEM	ETEST	blaKPC*	2	2	0	5	0.50	1.00
ETP	ETEST	blaKPC*, *blaCTX-M*	6	1	0	2	0.86	1.00
ETP	MicroScan	blaKPC*	2	7	0	0	0.22	NA
MEM	MicroScan	blaKPC*	2	7	0	0	0.22	NA
ETP	MicroScan	blaKPC*, *blaCTX-M*	6	3	0	0	0.67	NA

*SN* Sensitivity, *SP* Specificity, *GR* Genotype resistant, *GS* Genotype susceptible, *PR* Phenotype resistant, *PS* Phenotype susceptible

## Discussion

This study was a follow-up to previous work (Rose et al., submitted) in which we found that short-read only WGS-based AMR genotypes corresponded to phenotypic resistance to carbapenems (assessed using MicroScan) with only 78% sensitivity and 89% specificity for meropenem, and only 56% sensitivity for ertapenem (all isolates were non-susceptible). Our objectives here were to determine 1) whether long-read sequencing would impact AMR detection with respect to short-read only sequencing, and 2) if a second manual AMR phenotype assay (ETEST) would give the same phenotype outcomes as the automated MicroScan platform. Our overall goal was to investigate the reliability and robustness of WGS for clinical applications.

As expected, the short-read-only assemblies resulted in many more (*n* = 44 – 132) contigs per isolate, the longest of which was still only 20% of the expected chromosome size. In contrast, the long-read assembly methods resulted in a circular chromosomal contig in almost all isolates (the exception being the *N* + *I[I]* method for OB0019), and chromosomal contigs were of similar length among methods. Most of the variation among long-read methods was in the extra-chromosomal contigs, ranging in number from 1 – 10.

Several of our findings suggested that the fully hybrid *N* + *I[I]* method was optimal. First, in five isolates, all long-read methods resulted in the same number of contigs, although in four isolates, one of the contigs appeared to be duplicated/triplicated in the *N* and *N[I]* methods as compared to the *N* + *I[I]* method. This observation has been noted as an issue for long-read only sequencing [33]. In the other four isolates, the number of contigs differed between long-read methods, where three isolates had 1–2 additional contigs with the *N* and *N[I]* methods, and one isolate had an additional contig in the *N* + *I[I]* method. Additionally, the long-read only method (*N*) resulted in the fewest number of single copy complete genes compared to the other three methods. These results suggest that the fully hybrid method showed the best performance, while the long-read-only method performed the poorest among the long-read methods. These findings are similar to those reported in previous studies that compared sequencing platforms for *Pseudomonas aeruginosa* [27], *Haemophilus parasuis* [18], *Enterobacterales* [30], and *E. coli* [28].

With respect to AMR gene detection, a total of 109 gene/isolate combinations were detected by at least one of the four assembly methods. Of these, in five cases (8%), one of the methods failed to detect a gene that was detected by the other methods, either completely (*n* = 2) or with identity < 85% (*n* = 3). In all cases, either the short or long-reads-only methods failed to detect a gene, which further supports the use of hybrid methods. The concordance among sequencing platforms/assembly approaches is similar to other studies [13] though much higher than others [26].

On the other hand, we found that of the 109 gene/isolate combinations, 22 (20%) showed a different number of copies among assembly methods. In all 22 cases, the short-read-only method found only one copy, and in the majority of cases (77%) the fully hybrid method also identified a single copy. Only in one case did the fully hybrid method identify two copies of a gene while all other methods identified one copy. However, it is difficult to determine the true AMR gene copy numbers from these data alone. Surprisingly, even though the majority of the duplicated genes were found by the *N* and *N[I]* methods, none of these were localized to the extra-chromosomal contigs that were suspected to be falsely duplicated. In all cases, gene duplications found by the *N* + *I[I]* method were localized to circular contigs (which might be more robust), while one or both of the copies found by the *N* and *N[I]* methods were often (though not always) on linear contigs. Given the potential importance of using AMR genotypes in clinical settings, additional studies are needed to resolve this issue.

We also found that, while the two phenotyping assays used in this study were 100% concordant for the non-carbapenem drugs, they were discordant in 7/20 (35%) cases for the carbapenem drugs. This is somewhat distressing since carbapenems are essential drugs [20], and accurately predicting the susceptibility of a patient’s infection is critical for antimicrobial stewardship and improved clinical outcomes. Since MicroScan showed non-susceptibility while the ETEST showed susceptibility in all instances of the discordant phenotype results, it is possible that isolate quality (e.g. isolate age or storage conditions) may have impacted these results, since isolates were sub-cultured and tested with the ETEST in some cases several years after the MicroScan test. On the other hand, no issues with isolate quality were noted in any other context, and the patient with the most discordant results (P111) was also the most recently seen subject (in 2021). It is more likely that the subsequent subculturing of the isolates from frozen stocks with non-selective media resulted in the loss of both the *blaKPC-2* gene and the associated carbapenem resistance between the time of collection/MicroScan assay and the subsequent ETEST/sequencing. Ideally, all assays and sequencing experiments would have been performed on a single cultured colony, and/or a third phenotype assay would be used to resolve discrepancies; unfortunately, this was not possible in this retrospective study.

We also observed a discordance between AMR genotype and phenotype: although there was 100% concordance between AMR genotype and phenotype for all four non-carbapenem drugs, the presence/absence of the *blaKPC-2* showed very poor (≤ 50%) sensitivity for either ertapenem or meropenem in both phenotype assays, including the most recently performed ETEST. Other studies have generally found high concordance between meropenem resistance and presence of a carbapenemase gene [13, 15], so the lack of concordance here was somewhat surprising. Since the presence of an ESBL gene (e.g. *blaCTX-M-15*) may reduce susceptibility to ertapenem [34], we also considered the combination of *blaKPC-2* and *blaCTX-M-15,* which did increase sensitivity to ertapenem (86% and 67% for the ETEST and MicroScan, respectively) but did not fully account for the resistance patterns observed. Other mechanisms can reduce susceptibility to carbapenems [19], including mutations in the porin genes; however, these mutations were not detected after additional testing with AMRFinder using the species-specific option to detect point mutations (data not shown). These findings might suggest that AMR gene databases are incomplete, such that additional genes not yet discovered also confer some degree of resistance, or that combined mechanisms of resistance of genes may play a role. Additionally, different carbapenemase gene variants and/or differential gene expression may confer different degrees of susceptibility, which are not accounted for in a simple present/absent analysis.

An important limitation of this study, and many others, is the lack of data on patient treatment plans and the eventual clinical outcome, and therefore the inability to thoroughly understand the impact and importance of AMR gene detection and/or phenotype assays for patient care. Although studies that include these factors are costly and present administrative hurdles, they will be required to advance WGS-based analysis into clinical settings.

## Conclusions

Overall, these results, while from a small dataset, are encouraging in that all sequencing methods resulted in a similar ability to detect plasmids and AMR genes for three different *Enterobacterales* species. Outstanding issues to be resolved include determining which approach gives the most accurate copy number for AMR genes, the impact of copy number on phenotypic resistance, and whether multiple copies of an AMR gene are expressed at the same level. The incomplete correlation between AMR genes and carbapenem drugs in this dataset is concerning and may point to other mechanisms of resistance. Nonetheless, we have demonstrably shown that the lack of detection of a carbapenemase gene is not a result of sequence platform and/or assembly method.

## Methods

### Bacterial isolates

The clinical isolates used in this study were collected from three patients (P100, P107, P111) seen in the Ochsner Health network in the New Orleans metropolitan area from 2017 – 2021. For each patient, samples were collected over time and/or from different anatomical locations (Table 2). Cultures were processed by the clinical microbiology laboratory using standard protocols. Isolates were identified at the time of collection by matrix-assisted laser desorption/ionization time-of-flight mass spectrometry (MALDI-TOF MS; Bruker Daltonics, Billerica, MA) as *E. cloacae* complex (P100), *K. pneumoniae* (P107), and *K. aerogenes* (P111). The isolates were stored in Columbia broth with 20% glycerol at -70°C.

**Table 2:** Samples

Patient ID	Species	Sample ID	DPFS^a^	Tissue	Year of Collection
100	*Enterobacter cloacae complex*	OB0001	0	Blood	2017
		OB0002	6	Wound	2017
		OB0003	23	Blood	2017
107	*Klebsiella pneumonaie*	OB0019	72	Tissue	2018
		OB0020	80	Wound (1)	2018
		OB0021	80	Wound (2)	2018
111	*Klebsiella aerogenes*	OB0028	0	Wound	2021
		OB0029	10	Wound	2021
		OB0030	24	Wound	2021

^a^Days Post First Sample

### AMR phenotyping

Two phenotyping assays were used: 1) at the time of collection (2017 – 2021), minimum inhibitory concentrations (MICs) were determined by the MicroScan WalkAway plus System (Beckman Coulter Inc., Brea, CA); and 2) in October 2022, MIC values were determined using the ETEST (bioMérieux, Marcy-l'Étoile, France), using sub-cultured isolates from frozen stocks. All MICs were interpreted following the 2022 Clinical and Laboratory Standards Institute (CSLI) guidelines for interpretation and cutoff values.

### WGS generation and assembly

In 2021, bacterial isolates were sub-cultured from frozen stocks by plate streaking and overnight incubation at 37°C. Single colonies were selected from streaked plates and cultured overnight at 37°C in 2ml Luria broth growth media with constant shaking at 250RPM. Bacteria in 500µL broth were pelleted by centrifugation and broth supernatant removed. Genomic DNA was extracted from pellets using DNeasy Blood & Tissue Kit (Qiagen) with a species-appropriate lysis step. The DNA concentration was measured using an Invitrogen Qubit Fluorometer (ThermoFisher Scientific). For Illumina sequencing, purified DNA was used as input for Illumina library prep and sequencing on a NextSeq2000 sequencing machine generating paired-end reads of 150 bp. For ONT sequencing, the same purified DNA was used for PCR-free ligation library preparation using V14 chemistry. ONT sequencing was performed on a GridION platform using R10.4.1 flowcells. Basecalling was performed using Guppy (version 5.0.16) in super high accuracy mode and rejecting failed reads.

**Fig. 5:**
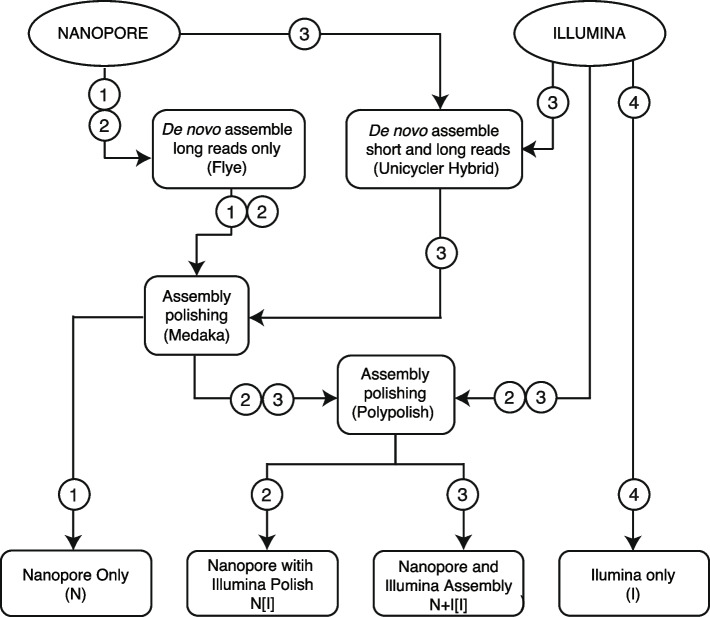
Flowchart showing four different assembly approaches for long and short read sequence data

Four different assembly pipelines, used to align reads to produce longer contiguous sequences (contigs), were compared (Fig. 5). The first was a long-read-only assembly (denoted as “*N*”). Next, we used two different hybrid approaches, one using a long-read-only assembly followed by a short-read polish (“*N[I]*”), and the second using a hybrid long- and short-read assembly with short-read polish (“*N* + *I[I]*”). Finally, we used a short-read only assembly (“*I*”). Based on other studies that compared different assembly methods [27, 30], and suggested protocols (https://github.com/rrwick/Trycycler/wiki/Guide-to-bacterial-genome-assembly) we chose to use Flye [35] for assembling long reads only, Medaka for long-read polishing [https://github.com/nanoporetech/medaka]), Polypolish [33] for short-read polishing of long-read assemblies, and Unicycler for both hybrid long- and short-read assembly and short-read-only assembly [36]. Assembly genome coverage was determined by contig circularization as determined by Flye/Unicycler, and gene completion assessed with Benchmarking Universal Single-Copy Orthologs (BUSCO) using the *Enterobacterales* database [37]).

### AMR/Plasmid identification

AMR genotypes were assessed using AMRFinderPlus (AMRF) [38, 39], which uses the NCBI Bacterial Antimicrobial Resistance Reference Gene Database. Genes with ≥ 85% coverage of the reference gene were considered present [12]. Genotype–phenotype associations were determined using annotation by AMRF. Putative plasmids were identified from polished contigs using PlasmidFinder [40], using the incompatibility (Inc) typing scheme based on replicon sequences.

### Supplementary Information

**Additional file 1.**

## Data Availability

Raw sequence data is available in the NCBI Short Read Archive under BioProject PRJNA939143.
