# Supplementary material for: Comparing antimicrobial resistant genes and phenotypes across multiple sequencing platforms and assays for Enterobacterales clinical isolates
Source: BMC Microbiol. 2023 Aug 18;23:225. doi: 10.1186/s12866-023-02975-x (PMC10436404; doi:10.1186/s12866-023-02975-x)
Supplement: Supplementary file 1 — Additional file 1. [file 12866_2023_2975_MOESM1_ESM.doc]

**Supplemental Table 1. Number of raw reads/nucleotides generated for nine samples using Illumina (short read) platform and the ONT (long read) platform.**

| **Technology** | **Sample** | **Reads** | **Nucleotides** | **Coverage** |
| --- | --- | --- | --- | --- |
| Illumina | OB0001 | 3,471,780 | 510,795,117 | 102 |
|  | OB0002 | 4,920,640 | 713,456,215 | 143 |
|  | OB0003 | 5,105,514 | 740,574,820 | 148 |
|  | OB0019 | 5,206,614 | 728,028,468 | 146 |
|  | OB0020 | 5,392,220 | 777,179,465 | 155 |
|  | OB0021 | 4,978,808 | 709,639,403 | 142 |
|  | OB0028 | 5,449,620 | 793,578,966 | 159 |
|  | OB0029 | 5,371,048 | 767,201,948 | 153 |
|  | OB0030 | 5,581,196 | 805,997,232 | 161 |
| ONT | OB0001 | 351,217 | 2,006,230,865 | 401 |
|  | OB0002 | 426,375 | 1,800,663,643 | 360 |
|  | OB0003 | 245,225 | 1,207,642,491 | 242 |
|  | OB0019 | 113,437 | 941,191,281 | 188 |
|  | OB0020 | 128,055 | 1,056,070,020 | 211 |
|  | OB0021 | 110,282 | 772,381,725 | 154 |
|  | OB0028 | 314,609 | 1,118,570,839 | 224 |
|  | OB0029 | 706,169 | 2,170,373,579 | 434 |
|  | OB0030 | 902,811 | 2,043,149,616 | 409 |

**Supplemental Table 2.**

| **Method** | **Sample** | **# Contigs** | **# Circularized contigs** | **Length longest contig**  **(bp)** |
| --- | --- | --- | --- | --- |
| *I* | OB0001 | 129 | 0 | 313,527 |
|  | OB0002 | 132 | 2 | 313,527 |
|  | OB0003 | 128 | 1 | 313,527 |
|  | OB0019 | 121 | 1 | 324,025 |
|  | OB0020 | 111 | 1 | 324,698 |
|  | OB0021 | 94 | 1 | 375,511 |
|  | OB0028 | 44 | 1 | 683,268 |
|  | OB0029 | 42 | 1 | 683,268 |
|  | OB0030 | 44 | 1 | 683,268 |
| *N* | OB0001 | 8 | 2 | 4,737,616 |
|  | OB0002 | 10 | 3 | 4,737,467 |
|  | OB0003 | 4 | 4 | 4,738,769 |
|  | OB0019 | 6 | 3 | 5,382,848 |
|  | OB0020 | 4 | 4 | 5,383,628 |
|  | OB0021 | 3 | 3 | 5,383,641 |
|  | OB0028 | 2 | 2 | 5,373,465 |
|  | OB0029 | 2 | 2 | 5,373,440 |
|  | OB0030 | 2 | 2 | 5,373,404 |
| *N[I]* | OB0001 | 8 | 2 | 4,737,465 |
|  | OB0002 | 10 | 3 | 4,737,306 |
|  | OB0003 | 4 | 4 | 4,738,624 |
|  | OB0019 | 6 | 3 | 5,382,596 |
|  | OB0020 | 4 | 4 | 5,383,370 |
|  | OB0021 | 3 | 3 | 5,383,355 |
|  | OB0028 | 2 | 2 | 5,373,302 |
|  | OB0029 | 2 | 2 | 5,373,301 |
|  | OB0030 | 2 | 2 | 5,373,281 |
| *N+I[I]* | OB0001 | 7 | 3 | 4,737,308 |
|  | OB0002 | 8 | 5 | 4,737,308 |
|  | OB0003 | 5 | 4 | 4,738,561 |
|  | OB0019 | 4 | 3 | 5,382,537 |
|  | OB0020 | 4 | 4 | 5,383,369 |
|  | OB0021 | 3 | 3 | 5,383,317 |
|  | OB0028 | 2 | 2 | 5,371,660 |
|  | OB0029 | 2 | 2 | 5,373,301 |
|  | OB0030 | 2 | 2 | 5,373,281 |

**Supplemental Table 3. BUSCO gene completion.**

| **Sample** | **Assembly Method** | **C** | **CS** | **CD** | **F** | **M** |
| --- | --- | --- | --- | --- | --- | --- |
| OB0001 | *I* | 437 | 436 | 1 | 0 | 3 |
|  | *N* | 434 | 433 | 1 | 0 | 6 |
|  | *N[I]* | 437 | 436 | 1 | 0 | 3 |
|  | *N+I[I]* | 437 | 436 | 1 | 0 | 3 |
| OB0002 | *I* | 437 | 436 | 1 | 0 | 3 |
|  | *N* | 430 | 429 | 1 | 2 | 8 |
|  | *N[I]* | 437 | 436 | 1 | 0 | 3 |
|  | *N+I[I]* | 437 | 436 | 1 | 0 | 3 |
| OB0003 | *I* | 437 | 436 | 1 | 0 | 3 |
|  | *N* | 435 | 434 | 1 | 1 | 4 |
|  | *N[I]* | 437 | 436 | 1 | 0 | 3 |
|  | *N+I[I]* | 437 | 436 | 1 | 0 | 3 |
| OB0019 | *I* | 434 | 431 | 3 | 1 | 5 |
|  | *N* | 429 | 426 | 3 | 5 | 6 |
|  | *N[I]* | 434 | 431 | 3 | 1 | 5 |
|  | *N+I[I]* | 434 | 431 | 3 | 1 | 5 |
| OB0020 | *I* | 434 | 431 | 3 | 1 | 5 |
|  | *N* | 427 | 424 | 3 | 7 | 6 |
|  | *N[I]* | 434 | 431 | 3 | 1 | 5 |
|  | *N+I[I]* | 434 | 431 | 3 | 1 | 5 |
| OB0021 | *I* | 434 | 431 | 3 | 1 | 5 |
|  | *N* | 427 | 424 | 3 | 5 | 8 |
|  | *N[I]* | 434 | 431 | 3 | 1 | 5 |
|  | *N+I[I]* | 428 | 425 | 3 | 5 | 7 |
| OB0028 | *I* | 436 | 434 | 2 | 0 | 4 |
|  | *N* | 426 | 424 | 2 | 7 | 7 |
|  | *N[I]* | 436 | 434 | 2 | 0 | 4 |
|  | *N+I[I]* | 436 | 434 | 2 | 0 | 4 |
| OB0029 | *I* | 436 | 434 | 2 | 0 | 4 |
|  | *N* | 429 | 427 | 2 | 6 | 5 |
|  | *N[I]* | 436 | 434 | 2 | 0 | 4 |
|  | *N+I[I]* | 436 | 434 | 2 | 0 | 4 |
| OB0030 | *I* | 436 | 434 | 2 | 0 | 4 |
|  | *N* | 429 | 427 | 2 | 5 | 6 |
|  | *N[I]* | 436 | 434 | 2 | 0 | 4 |
|  | *N+I[I]* | 436 | 434 | 2 | 0 | 4 |

C = complete; CS = complete single copy; CD = complete duplicated copy; F = fragmented; M = missing. Assembly methods are defined in the text.

**Supplemental Table 4. Contig number (as shown in Figure 1) on which AMR genes were found by three long-read assembly methods.**

| Sample | Method | *aac(3)-IIe* | *aadA1* | *aadA16* | *aph(3'')-Ib* | *aph(6)-Id* | *aac(6')-Ib-cr5* | *ampC* | *blaSHV-11* | *blaTEM-1* | *blaKPC-2* | *blaCTX-M-15* | *blaOXA-1* | *fosA5* | *fosA9* | *catA1* | *oqxA* | *oqxB9* | *oqxB19* | *qnrB1* | *qnrB6* | *arr-3* | *sul1* | *sul2* | *dfrA14* | *dfrA27* |
| --- | --- | --- | --- | --- | --- | --- | --- | --- | --- | --- | --- | --- | --- | --- | --- | --- | --- | --- | --- | --- | --- | --- | --- | --- | --- | --- |
| OB0001 | *N* | 3 | 2 |  | 3 | 3 | 3 |  |  | 3 |  | 3 | 3 |  |  | 2 | 1 | 1 |  | 6 |  |  |  | 3 | 6 |  |
|  | *N[I]* | 3 | 2 |  | 3 | 3 | 3 |  |  | 3 |  | 3 | 3 |  |  | 2 | 1 | 1 |  | 6 |  |  |  | 3 | 6 |  |
|  | *N+I[I]* | 2 | 2 |  | 2 | 2 | 2 |  |  | 2 |  | 2 | 2 |  |  | 2 | 1 | 1 |  | 2 |  |  |  | 2 | 2 |  |
| OB0002 | *N* | 3/7 | 2 |  | 3/7 | 3/7 | 3/7 |  |  | 3/7 |  | 3/7 | 3/7 |  |  | 2 | 1 | 1 |  | 6 |  |  |  | 3/7 | 6 |  |
|  | *N[I]* | 3/7 | 2 |  | 3/7 | 3/7 | 3/7 |  |  | 3/7 |  | 3/7 | 3/7 |  |  | 2 | 1 | 1 |  | 6 |  |  |  | 3/7 | 6 |  |
|  | *N+I[I]* | 2 | 2 |  | 2 | 2 | 2 |  |  | 2 |  | 2 | 2 |  |  | 2 | 1 | 1 |  | 2 |  |  |  | 2 | 2 |  |
| OB0003 | *N* | 2/3 | 2 |  | 2/3 | 2/3 | 2/3 |  |  | 2/3 |  | 2/3/4 | 2/3 |  |  | 2 | 1 | 1 |  | 2 |  |  |  | 2/3 | 2 |  |
|  | *N[I]* | 2/3 | 2 |  | 2/3 | 2/3 | 2/3 |  |  | 2/3 |  | 2/3/4 | 2/3 |  |  | 2 | 1 | 1 |  | 2 |  |  |  | 2/3 | 2 |  |
|  | *N+I[I]* | 2 | 2 |  | 2 | 2 | 2 |  |  | 2 |  | 2/3 | 2 |  |  | 2 | 1 | 1 |  | 2 |  |  |  | 2 | 2 |  |
| OB0019 | *N* | 2 |  | 3 |  |  | 2/3 |  | 1 | 2 | 3 | 2 | 2 | 1 |  |  | 1 | 1 |  | 2 | 5 | 3 | 6 |  | 2 | 3 |
|  | *N[I]* | 2 |  | 3 |  |  | 2/3 |  | 1 | 2 | 3 | 2 | 2 | 1 |  |  | 1 | 1 |  | 2 | 5 | 3 | 6 |  | 2 | 3 |
|  | *N+I[I]* | 2 |  | 3 |  |  | 2/3 |  | 1 | 2 | 3 | 2 | 2 | 1 |  |  | 1 | 1 |  | 2 | 3 | 3 | 3(x2) |  | 2 | 3 |
| OB0020 | *N* | 2 |  | 3 |  |  | 2/3 |  | 1 | 2 | 3 | 2 | 2 | 1 |  |  | 1 | 1 |  | 2 | 3 | 3 | 3(x2) |  | 2 | 3 |
|  | *N[I]* | 2 |  | 3 |  |  | 2/3 |  | 1 | 2 | 3 | 2 | 2 | 1 |  |  | 1 | 1 |  | 2 | 3 | 3 | 3(x2) |  | 2 | 3 |
|  | *N+I[I]* | 2 |  | 3 |  |  | 2/3 |  | 1 | 2 | 3 | 2 | 2 | 1 |  |  | 1 | 1 |  | 2 | 3 | 3 | 3(x2) |  | 2 | 3 |
| OB0021 | *N* | 2 |  |  |  |  | 2 |  | 1 | 2 |  | 2 | 2 | 1 |  |  | 1 | 1 |  | 2 |  |  |  |  | 2 |  |
|  | *N[I]* | 2 |  |  |  |  | 2 |  | 1 | 2 |  | 2 | 2 | 1 |  |  | 1 | 1 |  | 2 |  |  |  |  | 2 |  |
|  | *N+I[I]* | 2 (x2) |  |  |  |  | 2 |  | 1 | 2 |  | 2 | 2 | 1 |  |  | 1 | 1 |  | 2 |  |  |  |  | 2 |  |
| OB0028 | *N* |  |  |  |  |  |  | 1 |  |  |  |  |  |  | 1 |  | 1 | 1 |  |  |  |  |  |  |  |  |
|  | *N[I]* |  |  |  |  |  |  | 1 |  |  |  |  |  |  | 1 |  | 1 | 1 |  |  |  |  |  |  |  |  |
|  | *N+I[I]* |  |  |  |  |  |  | 1 |  |  |  |  |  |  | 1 |  | 1 | 1 |  |  |  |  |  |  |  |  |
| OB0029 | *N* |  |  |  |  |  |  | 1 |  |  |  |  |  |  | 1 |  |  | 1 |  |  |  |  |  |  |  |  |
|  | *N[I]* |  |  |  |  |  |  | 1 |  |  |  |  |  |  | 1 |  | 1 | 1 |  |  |  |  |  |  |  |  |
|  | *N+I[I]* |  |  |  |  |  |  | 1 |  |  |  |  |  |  | 1 |  | 1 | 1 |  |  |  |  |  |  |  |  |
| OB0030 | *N* |  |  |  |  |  |  | 1 |  |  |  |  |  |  | 1 |  | 1 | 1 |  |  |  |  |  |  |  |  |
|  | *N[I]* |  |  |  |  |  |  | 1 |  |  |  |  |  |  | 1 |  | 1 | 1 |  |  |  |  |  |  |  |  |
|  | *N+I[I]* |  |  |  |  |  |  | 1 |  |  |  |  |  |  | 1 |  | 1 | 1 |  |  |  |  |  |  |  |  |

**Supplemental Table 5. Plasmid localization and identity to reference.**

| **Sample** | **Method** | **Col 440II** | **Col (pHAD28)** | **Col RNAI** | **IncF IB(K)** | **IncF II(pKPX1)** | **IncH I2** | **IncH I2A** | **IncN** | **pKP1433** |
| --- | --- | --- | --- | --- | --- | --- | --- | --- | --- | --- |
| OB0001 | Illumina |  |  |  | 99 | 100 | 100 | 100 |  | 100 |
|  | Nanopore |  | 93 |  | 99 (#4) | 100 (#3) | 100 (#2) | 100 (#2) |  | 100 (#2) |
| OB0002 | Illumina |  |  |  | 99 | 100 | 100 | 100 |  | 100 |
|  | Nanopore |  | 93 |  | 99 (#4) | 100 (#3) | 100 (#2) | 100 (#2) |  | 100 (#2) |
| OB0003 | Illumina |  |  |  | 99 | 100 | 100 | 100 |  | 100 |
|  | Nanopore |  | 93 |  | 99 (#3) | 100 (#2) | 100 (#2) | 100 (#2) |  | 100 (#4) |
| OB0019 | Illumina | 100 |  |  | 99 |  |  |  | 100 |  |
|  | Nanopore |  | 92 |  | 99 (#2) |  |  |  | 100 (#3) |  |
| OB0020 | Illumina | 100 |  |  | 99 |  |  |  | 100 |  |
|  | Nanopore | 100 (#4) | 92 |  | 99 (#2) |  |  |  | 100 (#3) |  |
| OB0021 | Illumina | 100 |  |  | 99 |  |  |  |  |  |
|  | Nanopore | 100 (#3) |  |  | 99 (#2) |  |  |  |  |  |
| OB0028 | Illumina |  |  | 100 |  |  |  |  |  |  |
|  | Nanopore |  |  | 100 (#2) |  |  |  |  |  |  |
| OB0029 | Illumina |  |  | 100 |  |  |  |  |  |  |
|  | Nanopore |  |  | 100 (#2) |  |  |  |  |  |  |
| OB0030 | Illumina |  |  | 100 |  |  |  |  |  |  |
|  | Nanopore |  |  | 100 (#2) |  |  |  |  |  |  |

The contig number on which the plasmid was found is noted in parentheses for Nanopore assemblies.
